# Utilization of a Sugarcane100K Single Nucleotide Polymorphisms Microarray-Derived High-Density Genetic Map in Quantitative Trait Loci Mapping and Function Role Prediction of Genes Related to Chlorophyll Content in Sugarcane

**DOI:** 10.3389/fpls.2021.817875

**Published:** 2021-12-28

**Authors:** Guilong Lu, Yong-Bao Pan, Zhoutao Wang, Fu Xu, Wei Cheng, Xinge Huang, Hui Ren, Chao Pang, Youxiong Que, Liping Xu

**Affiliations:** ^1^Key Laboratory of Sugarcane Biology and Genetic Breeding, Ministry of Agriculture and Rural Affairs, Fujian Agriculture and Forestry University, Fuzhou, China; ^2^Institute of Vegetables, Tibet Academy of Agricultural and Animal Husbandry Sciences, Lhasa, China; ^3^Sugarcane Research Unit, United States Department of Agriculture (USDA), Agricultural Research Service (ARS), Houma, LA, United States

**Keywords:** sugarcane, SNP chip, chlorophyll content, QTL mapping, candidate gene prediction

## Abstract

Chlorophyll is the most important pigment for plant photosynthesis that plays an important role in crop growth and production. In this study, the chlorophyll content trait was explored to improve sugarcane yield. Two hundred and eighty-five F_1_ progenies from the cross YT93-159 × ROC22 with significantly different chlorophyll contents were included as test materials. The chlorophyll content of the +1 leaves during elongation phase was measured using a SPAD-502 meter through a three-crop cycle (plant cane, first ratoon, and second ratoon). Linkage analysis was conducted on a high-density genetic map constructed based on the sugarcane 100K SNP chip. In addition, Fv/Fm, plant height, stalk diameter, brix data were collected on plant cane during the elongation and maturation phases. The results showed that the +1 leaf SPAD values, which can be used as an important reference to evaluate the growth potential of sugarcane, were significantly and positively correlated with the Fv/Fm during elongation phase, as well as with plant height, stalk diameter, and brix during maturity phase (*P* < 0.01). The broad sense heritability (*H*^2^) of the chlorophyll content trait was 0.66 for plant cane crop, 0.67 for first ratoon crop, and 0.73 for second ratoon crop, respectively, indicating that this trait was mainly controlled by genetic factors. Thirty-one quantitative trait loci (QTL) were detected by QTL mapping. Among them, a major QTL, *qCC-R1*, could account for 12.95% of phenotypic variation explained (PVE), and the other 30 minor QTLs explained 2.37–7.99% PVE. Twenty candidate genes related to chlorophyll content were identified in the QTLs plus a 200-Kb extension region within either sides, of which four were homologous genes involved in the chlorophyll synthesis process and the remaining 16 played a certain role in chlorophyll catabolic pathway, chloroplast organization, or photosynthesis. These results provide a theoretical reference for analyzing the genetic mechanism of chlorophyll synthesis and subsequent improvement of photosynthetic characteristics in sugarcane.

## Introduction

Sugarcane (*Saccharum* spp. hybrids), which can ratoon for several years (Xu et al., 2021), is one of the most promising industrial crops in the world. It is widely cultivated in more than 100 countries or regions in tropical and subtropical regions and provides 80% of the world’s sugar and 60% of bioethanol, and a total economic value of 75 billion US dollars (FAO, 2019). In addition, the energy output-to-input ratio for ethanol production by sugarcane is five times higher than maize (Goldemberg, 2008; Waclawovsky et al., 2010). Sugarcane by-products are also valuable (Sindhu et al., 2016). China is the world’s third largest sugarcane producer and the largest sugar importer. Its self-sufficiency rate of sugar is only about 65% and about four to five million tons of sugar are imported every year in order to meet the national demands. While the world’s human population is expected to exceed 9.7 billion by 2050 (United Nations [UN], 2019), the demand for energy and food will increase by at least 25% according to the current per capita consumption standard. In response to frequent extreme weather and limited arable land resources, it is particularly necessary and urgent to increase the unit yield of sugarcane and other crops. Although the theoretical fresh cane yield of sugarcane can reach more than 380 t/ha (Waclawovsky et al., 2010), the current cane yields are about 77 t/ha and 73 t/ha in China and world, respectively (FAO, 2019), leaving a lot of rooms for improvement.

The photosynthesis is a prerequisite for the accumulation of crop yields. Increasing yields by improving photosynthetic characteristics has gradually become a new hotspot for crop breeding (Raines, 2011; Ort et al., 2015). Chlorophyll is the most important photosynthetic pigment for plants to absorb, transmit and convert light energy, and plays a vital role in the growth and development of crops (Melkozernov and Blankenship, 2006; Tanaka and Tanaka, 2006). Many studies have shown that leaf chlorophyll content is closely related to the biological yield and economic gain (Mauromicale et al., 2006; Quarrie et al., 2006; Gao et al., 2013; Faralli and Lawson, 2020). Leaf chlorophyll content has been used to evaluate crop drought tolerance (Li et al., 2006; Gholamin and Khayatnezhad, 2020) and to predict aboveground biomass yield (Liu et al., 2019). Moreover, within a certain range, the photosynthetic rate of crops rises along with increasing chlorophyll content (Mae, 1997; Guo et al., 2008; Nahakpam, 2017; Zhao et al., 2019). Variation in chlorophyll content is mainly controlled by the expression of genes related to chlorophyll biosynthesis (Lee et al., 2005; Wang et al., 2008, 2020; Sakuraba et al., 2013). Hence, a better understanding of the genetic basis of chlorophyll content may help accelerate high-yield crop breeding.

In earlier studies, the researchers mainly used quantitative trait locus (QTL) linkage analysis or genome-wide association study (GWAS) to dissect the genetic basis of chlorophyll content variation. For example, Pinto et al. (2010) measured the chlorophyll contents of 167 wheat recombined inbred lines (RIL) grown under three environments and genotyped these RILs with 74 single sequence repeat (SSR), 249 amplified fragment length polymorphism (AFLP), and 264 diversity array technology (DArT) molecular markers for QTL mapping. They detected five QTLs related to leaf chlorophyll content on chromosomes 1B, 1D, and 5A, which could explain 7.8–11.8% of chlorophyll content variation. Using 2-year data on chlorophyll a and chlorophyll b from a cabbage F_2:3_ population for QTL mapping, Ge et al. (2012) detected 10 QTLs, which explained 7–17% of the phenotypic variation. Ye et al. (2017) grew 132 rice RILs derived from the cross 93–11 × PA64S under two nitrogen application levels and found 32 major QTLs at two developmental stages. One QTL contributed 6.0–20.8% to the variation in the chlorophyll content of rice under low nitrogen conditions. Jian et al. (2020) re-sequenced 588 accessions of a Cole germplasm collection of *Brassica napus* and identified 385,692 high-quality single nucleotide polymorphisms (SNP) markers. Then they performed a GWAS analysis on chlorophyll content trait and identified 5 and 46 significant SNPs from 23 candidate genes. However, similar kind of study has never been reported on sugarcane.

SNP is the most promising marker after restriction fragment length polymorphism (RFLP) and SSR (Rafaiski, 2002; Shabrukov, 2016). When SNP combines with biochip technology, it provides a high-throughput screening platform for gene mining and marker discovery that are associated with agronomic and economic traits (Dalton-Morgan et al., 2014; McCouch et al., 2016; Zhang et al., 2019). However, the ploidy is an important factor that affects SNP marker identification and verification (Bassil et al., 2015). Until recent years, with the rapid development of high-throughput sequencing technology and cost reduction, SNP chips have been developed for polyploid crops one after another. These chips include Cole (*Brassica napus* L.; 2*n* = 4*x* = 48; genome size = ∼845 Mb) (Chalhoub et al., 2014) 60K array (Clarke et al., 2016), wheat (*Triticum asetivum* L.; 2*n* = 6*x* = 42; genome size = ∼ 14.5 Gb) (IWGSC et al., 2018) 820K array (Winfield et al., 2016), strawberry (*Fragaria ananassa* Duch.; 2*n* = 8*x* = 56; genome size = ∼698 Mb) (Hirakawa et al., 2014) 90K array (Bassil et al., 2015), sugarcane (*Saccharum* spp. hybrids; 2*n* = 12*x* = 100–130; genome size = ∼10 Gb) (Roach, 1989; D’Hont et al., 1998) 345K array (Aitken et al., 2017), and recently, 100K array (You et al., 2019). So far, the application of SNP chips has been mainly focused on disease resistance traits (Gao et al., 2016; Wu et al., 2016; You et al., 2019, 2020) with one exceptional case on chlorophyll content trait (Gao et al., 2021).

Our previous research showed that the leaf chlorophyll content might differ quite differently among the parental germplasms. Based on this observation, we made a cross between two popular major sugarcane cultivars YT93-159 and ROC22 and used their F_1_ progenies as a mapping population. We grew the population in the field for three consecutive years and collected phenotypic trait data from plant cane, first ratoon, and second ratoon crops. Then, we conducted linkage and QTL analyses based on two parents’ genetic maps constructed by the sugarcane 100K SNP chip (You et al., 2019). In addition, we mined the genes related to chlorophyll synthesis analyzed the correlation between leaf chlorophyll content and plant height, diameter, total soluble solid content (brix) and Fv/Fm. The results from this study are expected to provide a theoretical reference for the genetic improvement of sugarcane.

## Materials and Methods

### Plant Material and Field Experiment Design

In this study, 285 F_1_ progenies from the cross (YT93-159 × ROC22) and the parents were included in a mapping population. The cross was made in 2014 at the Hainan Sugarcane Breeding Station, Guangdong Provincial Bioengineering Institute (Guangzhou Sugarcane Industrial Research Institute). Two-bud stalk billets of the mapping population were planted in a field-testing site of Fujian Agriculture and Forestry University in early January 2019 using a random block design, single row plots, and three replications. The row length was 1.0 m and the row spacing was 1.2 m. Seven billets or 14 buds were planted in each row (plot). The annual nitrogen application rate was 300 kg urea/ha with rainfed precipitation and uniform field management.

### Phenotype Determination and Evaluation

From 2019 to 2021, an SPAD-502 Plus instrument (Konica Minolta Sensing Inc., Osaka, Japan) was used to measure the leaf chlorophyll content at the middle hypertrophic leaf (fully extended +1 leaf) visible at the top during mid-August each year. During the third quarter of August 2019, the maximum quantum yield of PSII, which represents the ratio between variable and maximum fluorescence of chlorophyll (Fv/Fm), were measured using an IMAGING-PAM fluorometer (Heinz Walz, Effltrich, Germany) on the same leaves. The leaf was wrapped in dark cloth for 20 min prior to the measurement. Five plants that grow normally were measured in each plot and each plant was measured three times. At the end of 2019, the brix, height, and stalk diameter (SD) were measured with a portable refractometer (PR-101α, ATAGO Inc., Japan), a standard meter, and an electronic Vernier caliper (MNT-150T, Meinaite Inc., Germany), respectively. Again, measurements were done on five plants per plot. The IBM SPSS^®^ V25 software (International Business Machines Inc., California, United States) was used for statistical and correlation analyses. Duncan’s new multiple range method (Yang et al., 2019) was used for significance testing. Broad sense heritability (*H*^2^) was estimated using the formula below:


$${\text{H}}^{2}=\sigma_{\text{g}}^{2}/\left(\sigma_{\text{g}}^{2}+\sigma_{\text{e}}^{2}\right)$$

Where $\sigma_{\text{g}}^{2}$ and *$\sigma_{\text{e}}^{2}$* are the estimation of the genotypic variance and error variance, respectively.

### Genetic Map Construction and Quantitative Trait Loci Analysis

The sugarcane 100K SNP chip was customized by Thermo Fisher Scientific (Massachusetts, United States) and we commissioned it to perform population genotyping. After screening for polymorphic markers, the isolated population genotype data was imported into GACD V1.2 software (Chinese Academy of Agricultural Sciences, Beijing, China) to construct a genetic linkage map [by our group (unpublished)]. Eighty-eight linkage groups were constructed for YT93-159 with 1,389 markers and a map length of 4,027.79 cM. Ninety linkage groups were constructed for ROC22 with 776 markers and a map length of 2,815.92 cM. Inclusive composite interval mapping (ICIM) was selected for QTL positioning with following parameters: mapping parameters step 1.0 cM, logarithm of odds (LOD) manual input 3.0, and other parameters default. The genotype data was from the linkage map construction and the phenotype data was the average value of each plot. Phenotypic variation explained (PVE) greater than or equal to 10% was defined as a major QTL. If less than 10%, it was assigned as a minor QTL. When the same QTL appeared more than one crop, it was considered a consistent QTL. Detected QTLs were named in italics fonts according to McCouch et al. (1997), starting by letter “q,” followed by corresponding trait and linkage group number. R studio software (R-Tools Technology Inc., Ontario, Canada) and Adobe Illustrator CS6 software (Adobe Systems Inc., California, United States) were used to assist in drawing the QTL distribution map on the linkage group.

### Candidate Gene Prediction and Analysis

Using the genome GFF3 sequence data of LA Purple, a *S. officinarum* accession,^1^ we searched for all genes located within the QTLs and 200 Kb sequence region in both sides of the QTL markers. *Saccharum officinarum* contributes more than 80% of the genomes of modern sugarcane cultivars (D’Hont et al., 1996). We also blasted the *Arabidopsis* genome with an *e*-value threshold of 1e-10.^2^ *Arabidopsis* genes with the highest homology were used in functional annotations. We also used the keyword “chlorophyll” to search for related candidate genes. Genomic locations of candidate genes were obtained from *S. officinarum* genome and set as the inputs to the TBtools V1.0986 software (Chen et al., 2020). Gene structures were depicted using an online tool GSDS V2.0.^3^

## Results

### Phenotypic Heritability and Correlation Evaluation

**FIGURE 1:**
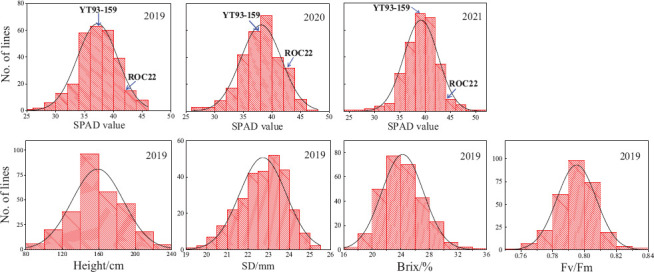
Frequency distribution of +1 leaves SPAD, plant height, stalk diameter, brix, and Fv/Fm in a sugarcane mapping population.

The values of SPAD, plant height, stalk diameter, brix, and Fv/Fm showed an obvious continuous normal distribution (Figure 1), suggesting that it is appropriate for QTL analysis. The results of Pearson correlation coefficient analysis of IBM SPSS V25 software are shown in Table 1. The SPAD values were significantly correlated with the values of plant height, stem diameter, brix, and Fv/Fm (*P* < 0.01). Moreover, the correlation among SPAD values of plant cane, first ratoon, and second ratoon crops was greater than 60% (*P* < 0.001). The *H*^2^ of the leaf chlorophyll content trait were 0.66, 0.67, and 0.73 for plant cane, first ratoon, and second ratoon crops, indicating that the leaf chlorophyll content trait of this mapping population was mainly determined by genetic factors.

**TABLE 1:** Correlation analysis of SPAD to plant height, stalk diameter, brix, and Fv/Fm in a sugarcane mapping population.

Year/Crop	2019/Plant cane				2020/First ratoon	2021/Second ratoon
Traits	Height	SD	Brix	Fv/Fm	SPAD	SPAD
SPAD (2019)	0.2071***	0.3588***	0.1849**	0.2364***	0.7264***	0.6148***
SPAD (2020)	–	–	–	–	–	0.6668***

*** and ** indicate significance at p < 0.001 and p < 0.01, respectively. “–” means undetermined.

### Phenotypic Analysis

The results of SPAD analysis on leaf chlorophyll contents of the two parents and their F_1_ mapping population during the three crop years is shown in Table 2. The male parent ROC22 had significantly higher SPAD values than the female parent YT93-159 did (*P* < 0.01). The mean SPAD values of the two parents increased slightly year by year, but the increase amount was not significant at *P < 0.05*, which indicates that the trait is relatively stable in the parents. The overall coefficient of variation of the F_1_ mapping population in three environments were 9.41, 9.33, and 8.62%, respectively. The leaf chlorophyll content of the F_1_ hybrid population varied over a wide range in every crop and there was an obvious bidirectional super-parental separation. It also showed the characteristics of a normal distribution (Figure 1), indicating that the leaf chlorophyll content trait conforms to the characteristics of quantitative traits controlled by polygenes.

**TABLE 2:** SPAD analysis on leaf chlorophyll contents of the two parents and F_1_ mapping population.

Trait	Environment	Parent		F_1_ mapping population		
		YT93-159	ROC22	Range	Mean ± SD	*CV*(%)
Leaf chlorophyll content	2019	37.40 B	42.16 A	24.66–45.43	37.22 ± 3.50	9.41
	2020	37.76 B	42.70 A	27.03–46.08	38.12 ± 3.56	9.33
	2021	38.75 B	44.23 A	25.78–51.58	39.17 ± 3.38	8.62

*Different capital letters indicate the significant difference between parents at level 0.01. SD, standard deviation; CV, coefficient of variation.*

### Quantitative Trait Loci Mapping

**FIGURE 2:**
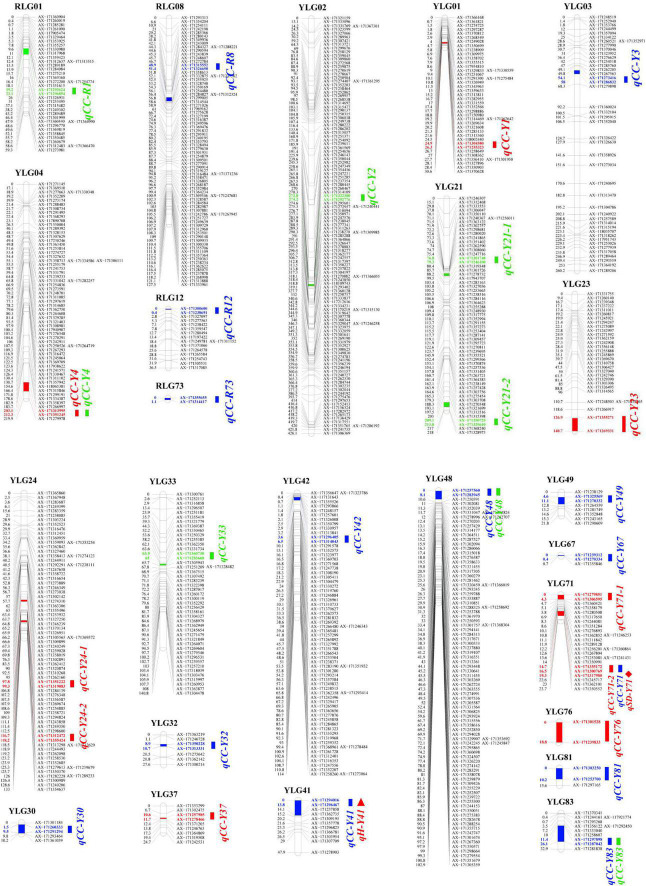
A QTL map for leaf chlorophyll content trait using a F_1_ mapping population of the cross (YT93-159 × ROC22). “CC” means chlorophyll content. “Y” represents parent YT93-159. “R” represents parent ROC22. Red name represents the plant cane crop in 2019. Blue name represents the first ratoon crop in 2020. Green name represents the second ratoon crop in 2021. Rectangle = chlorophyll content traits. Triangle = plant height traits. Rhombus = stalk diameter traits.

Based on a high-quality linkage map [by our group (unpublished)] and the combined SPAD values of the F_1_ mapping population, 9, 14, and 8 QTLs were detected for plant cane, first ratoon, and second ratoon crops, respectively. Of which, 27 QTLs were detected in YT93-159 and four QTLs in ROC22. It is interesting that only one major QTL, *qCC-R1*, which could explain 12.95% of the phenotypic variation, while the remaining 30 QTLs accounted for 2.37–7.99% of the phenotypic variation. Moreover, the genetic distance between the QTL peak and the nearest marker was mostly below 1.0 cM, with an average of only ∼0.8 cM (Supplementary Table 1). The specific positions of these 31 QTLs on the linkage groups are shown in Figure 2. It is worth mentioning that four QTLs, *qCC-Y4*, *qCC-Y48*, *2019-qCC-Y71-2* (*2020-qCC-Y71*) and *qCC-Y83*, were detected in two crop seasons on the YT93-159 map. In addition, we found that one QTL (*qH-Y41*, 7.20%) related to plant height completely overlapped with the QTL *qCC-Y41* for chlorophyll content traits, and there was a QTL (*qSD-Y71*, 4.75%) related to stem diameter closely adjacent to the QTL *2019-qCC-Y71-2* (*2020-qCC-Y71*) for chlorophyll content traits. However, no linked QTL of brix and Fv/Fm and chlorophyll content were observed.

### Candidate Gene Mining

**FIGURE 3:**
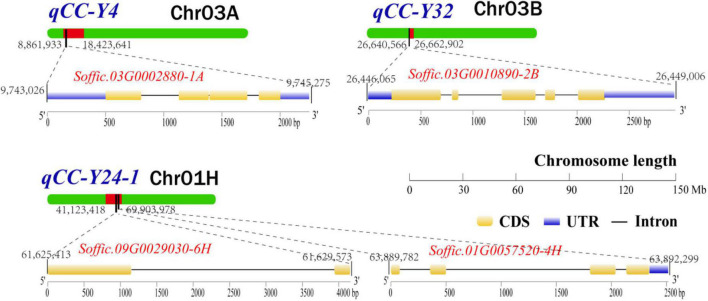
The genomic location, conserved domain, and gene structure of four major candidate genes involved in the chlorophyll synthesis pathway. UTR, untranslated region; CDS, coding sequence.

After removing the four duplicate QTLs, we searched and located the 27 QTLs in the genome of *S. officinarum* using the probe sequence information in Supplementary Table 2, and all the genes in the range of QTLs interval and the 200 Kb extension region were extracted. In reference to the annotation files of the *Arabidopsis* genome, we identified 20 candidate genes that were related to chlorophyll metabolism pathway (Supplementary Table 3). Eighteen candidate genes were located in QTL interval. Two candidate gene (*Soffic.03G0010890-2B* and *Soffic.04G0016360-1A*) was found in the 200 Kb extension region. In total, eight related genes were found in one major QTL and four repeated QTLs. Four genes (*Soffic.03G0002880-1A*, *Soffic.01G0057520-4H*, *Soffic.09G0029030-6H*, and *Soffic.03G0010890-2B*) participated in chlorophyll biosynthetic process. The other genes (*Soffic.01G0009920-2P*, *Soffic.01G0017050-4F*, *Soffic.01G0024550-4F*, and *Soffic.02G0025400-2P*) were involved in chlorophyll catabolic process. The remaining 12 candidate genes were involved either in photosynthesis or in chloroplast organization. Finally, the genomic location, conserved domain, and gene structure of four major candidate genes (*Soffic.03G0002880-1A*, *Soffic.03G0010890-2B*, *Soffic.09G0029030-6H*, and *Soffic.01G0057520-4H*) involved in the chlorophyll synthesis pathway are shown in Figure 3.

## Discussion

Leaf chlorophyll content is an important indicator for evaluating photosynthetic capacity and crop yield (Ghosh et al., 2003; Gitelson et al., 2014; Yu et al., 2019; Zhao et al., 2019). The chlorophyll content may change drastically both during plant development and in response to various stresses (Nahakpam, 2017; Ye et al., 2017; Bhusal et al., 2018; Sun et al., 2019; Gao et al., 2021). Sugarcane is a high-biomass perennial crop with a growth cycle of 10–14 months. The elongation period is critical for cane yield and sugar formation (Chen et al., 2010). To explore the environmental impact on the phenotypes, three consecutive crop cycles, i.e., plant cane, first ratoon, and second ratoon, with 12 months for each crop cycle, were chosen to conduct chlorophyll content measurements. Furthermore, the same management with equal amounts of fertilizers was strictly adopted for each crop cycle. In this study, a significant positive correlation was observed between the SPAD of +1 leaf chlorophyll content of and the Fv/Fm during the elongation period. Similar results were also found between the SPAD and the stalk diameter, plant height, or brix during harvest time (*P < 0.01*). These results are basically consistent with those from Luo et al. (2006); Chen et al. (2010), and Yang et al. (2015) and demonstrate that the leaf chlorophyll content can be used as an important reference for evaluating the growth potential of sugarcane.

Modern sugarcane varieties are highly heterozygous (Zhang et al., 2018) and phenotypic traits segregate widely among cross progenies. Since the F_1_ hybrid population can be asexually propagated, the population can be used permanently to study genetic inheritance (Asnaghi et al., 2004). Taking our F_1_ mapping population as an example, the leaf chlorophyll content segregated extensively and exhibited an obvious bidirectional super-parental distribution, typical characteristics of quantitative traits (Table 2 and Figure 1). Moreover, the average leaf SPAD value of the parents and the F_1_ population tended to increase slightly year by year, although the increase was not significant at *P* < 0.05. It is speculated that this may be due to the fact that sugarcane belongs to one of perennial grassy ratoon crops. In comparison to the plant cane, the first and second ratoon crops may have more developed and deeper roots. A good root system helps to improve water and nutrient absorption and is conducive to the synthesis and accumulation of chlorophylls, but it is less affected by the planting and ratooning seasons. The *H*^2^ of leaf chlorophyll content over 3 years varied from 0.66 to 0.73, which are slightly lower than the broad heritability of wheat heading, flowering, and flowering stages either under normal irrigation (0.74–0.81) or under drought stress (0.74–0.76) (Gao et al., 2021). However, the *H*^2^ was similar to the heritability of different leaf positions in corn 5 days after pollination (0.66–0.67) (Shi et al., 2019), demonstrating that the leaf chlorophyll content trait is relatively stable for locating QTLs.

The size of the mapping population and the density of molecular markers directly affect the accuracy and resolution power of the markers positioned (Beavis, 1994). Currently, the size of most mapping populations for sugarcane QTL research are about 100–200 involving AFLP, RFLP, or SSR markers (Yang, 2015; Singh et al., 2016; Balsalobre et al., 2017). Due to the lack of detection tools for large populations or high-density genotyping, the genetic distance between markers and genes targeted is relatively large (Daugrois et al., 1996; Raboin et al., 2006; Wang et al., 2018; Rahman et al., 2019). In this study, the Axiom Sugarcane100K SNP Array was used. It contains 100,097 low-dosage SNPs, of which 64,726 are single-dose and 35,371 are double dose (You et al., 2019). Furthermore, the analysis population includes a total of 285 progenies, which is significantly higher than previous studies (Yang, 2015; Singh et al., 2016; Balsalobre et al., 2017).

We were the first to analyze the genetic basis of the leaf chlorophyll content trait in sugarcane. We detected 31 QTLs in three consecutive crops, but only one of which is a major QTL (12.95%). However, 27 QTLs were detected on the YT93-159 map, which was significantly higher than 4 QTLs detected on the ROC22 map. This may be related to the quantity (1,389 SNP markers) and density (2.9 cM/marker) on the YT93-159 map vs. 776 SNP markers at 3.6 cM/marker on the ROC22 map (Supplementary Figure 1). Although four QTLs were consistently detected in two crops, no common QTL was detected among all three crop cycles. Unfortunately, due to the lack of a complete genome sequence of modern sugarcane cultivars (Garsmeur et al., 2018; Zhang et al., 2018), we were unable to map the two QTLs (*qCC-Y23* and *qCC-Y41*). In this study, the average distance of a marker closest to the QTL peak is 0.8 cM, which is similar to the brown rust (0.1 cM-8.1 cM) and orange rust (0.2 cM-2.2 cM) markers in sugarcane identified by Yang et al. (2017, 2018) based on a GBS genetic map. Furthermore, this study demonstrates the feasibility and reliability of locating QTL by using SNP chips.

The leaf chlorophyll content is mainly regulated by the expression of genes related to its synthesis. In order not to omit any related gene, we expanded the search range to 200 Kb on both sides of the QTL region. A total of 20 candidate genes were observed, and these genes are mainly located in the chloroplast envelope or/and stroma, thylakoid membrane. The number of candidate genes obtained is similar to the 23 genes reported by Jian et al. (2020) on *Brassica napus*. Three candidate genes, namely, *Soffic.03G0002880-1A*, *Soffic.02G0015390-2B*, and *Soffic.02G0026560-5H*, are equivalents of *AT1G78600*, *AT1G19150*, and *AT3G61470* in *Arabidopsis*, respectively. The three genes are involved de-etiolation (Chang et al., 2008), photo protection (Chen et al., 2018), and regulation of spectral properties of pigments (Wientjes et al., 2012), respectively, in *Arabidopsis*.

In this study, we also identified four genes from the chlorophyll synthesis pathway (Supplementary Table 3). *Soffic.03G0002880-1A* influences chloroplast biogenesis and function by encoding the light-regulated zinc finger protein 1 (LZF1) and regulating the expression of chloroplast protein-encoding genes (Chang et al., 2008). *Soffic.01G0057520-4H* encodes a hypothetical chloroplast open reading frame 54 protein (YCF54), and loss of this protein is accompanied with leaf chlorosis (Herbst et al., 2018). *Soffic.09G0029030-6H* encodes a NAD(P)-binding Rossmann-fold superfamily protein. The protein has 3,8-divinyl protochlorophyllide a 8-vinyl reductase (DVR) activity and is indispensable for monovinyl chlorophyll synthesis (Nagata et al., 2005). *Soffic.03G0010890-2B* encodes a dicarboxylate di-iron protein (Crd1), also known as CHL27. The protein is required for the synthesis of protochlorophyllide (Tottey et al., 2003). In addition, inactivation of *ATAB2*, an *Arabidopsis* gene homologous to *Soffic.04G0016360-1A*, strongly affects thylakoid membrane biogenesis and leads to an albino phenotype (Barneche et al., 2006). It is worth mentioning that *Soffic.04G0016360-1A* and *Soffic.03G0010890-2B* are found within the 200 Kb extended region, suggesting that more candidate genes may be found if the search region is extended further. The genes identified from this study are important reference for further understanding of the mechanism of chlorophyll synthesis in sugarcane.

In this study, we found that *qSD-Y71* (4.75%), a stalk diameter-related QTL marker, was closely linked to a QTL (*2019-qCC-Y71-2*, 5.05%) controlling the leaf chlorophyll content, which should be verified in future research. Although a plant height-related QTL (*qH-Y41*, 7.20%) completely overlapped with the chlorophyll content trait on the YT-LG41 linkage group, but unfortunately, the *qH-Y41* marker could not be aligned to any chromosome. Therefore, we were unable to locate a candidate gene. However, further advancement of sugarcane genome research may help improve the outcome of follow-up research. In this study, we did not identify any QTL marker that linked the leaf chlorophyll content trait to brix or Fv/Fm. Multi-trait linkage markers are valuable for further crop yield improvement, but in reality, it is very difficult to apply these markers in the breeding process of a crop like sugarcane.

Changes in leaf chlorophyll biosynthesis directly affect the intracellular chloroplast ultrastructure, leaf physiological and biochemical characteristics, and leaf color (Tanaka and Tanaka, 2006; Wang et al., 2016). A recent study by Gu et al. (2017) showed that the chlorophyll content of a radiation-induced rice mutant was 40–50% lower than its wild type Zhefu 802. However, this mutant had a much higher photosynthesis and photosynthetic N use efficiency (PNUE) than the wild type. Whether there were similar cases in sugarcane remains to be explored.

## Conclusion

Field experimental data were collected from three consecutive crops (plant cane, first ratoon, and second ratoon) of a sugarcane F_1_ mapping population consisting of 285 progenies. There were significant and positive correlations (*P* < 0.01) between the SPAD of +1 leaf chlorophyll content and the Fv/Fm during elongation period, as well as the stalk diameter, plant height and brix during harvest time (*P* < 0.01). In addition, a sugarcane 100K SNP Microarray-derived high-density genetic map was used to detect one major QTL (*qCC-R1*) and 30 minor QTLs associated with the leaf chlorophyll content. Furthermore, 20 candidate genes were identified within the QTLs and its 200 Kb extention region on either side according to an unpublished genome of LA Purple, a *Saccharum officinarum* accession. Four genes were involved in chlorophyll biosynthetic pathway and 16 genes were involved in chlorophyll catabolism, chloroplast organization, or photosynthesis processes. This study provides good information for further research on genetic improvement of leaf chlorophyll content in sugarcane.

## Data Availability Statement

The original contributions presented in the study are included in the article/Supplementary Material, further inquiries can be directed to the corresponding authors.

## Author Contributions

GL and LX conceptualized the study and designed the experiment. GL, Y-BP, LX, and YQ prepared the manuscript. LX provided the materials. GL, WC, FX, XH, HR, and CP performed the experiments. GL and ZW conducted the data analysis. All authors read and approved the manuscript.

## Conflict of Interest

The authors declare that the research was conducted in the absence of any commercial or financial relationships that could be construed as a potential conflict of interest.

## Publisher’s Note

All claims expressed in this article are solely those of the authors and do not necessarily represent those of their affiliated organizations, or those of the publisher, the editors and the reviewers. Any product that may be evaluated in this article, or claim that may be made by its manufacturer, is not guaranteed or endorsed by the publisher.

## Footnotes

1
http://sugarcane.zhangjisenlab.cn/sgd/html/index.html

2
https://www.arabidopsis.org/Blast/index.jsp

3
http://gsds.gao-lab.org/

## Supplementary Material

Supplementary Figure 1 Marker density of YT93-159 and ROC22 genetic linkage map.
