# Supplementary figures and images for: Utilization of a Sugarcane100K Single Nucleotide Polymorphisms Microarray-Derived High-Density Genetic Map in Quantitative Trait Loci Mapping and Function Role Prediction of Genes Related to Chlorophyll Content in Sugarcane
Source: Front Plant Sci. 2021 Dec 28;12:817875. doi: 10.3389/fpls.2021.817875 (PMC8750863; doi:10.3389/fpls.2021.817875)

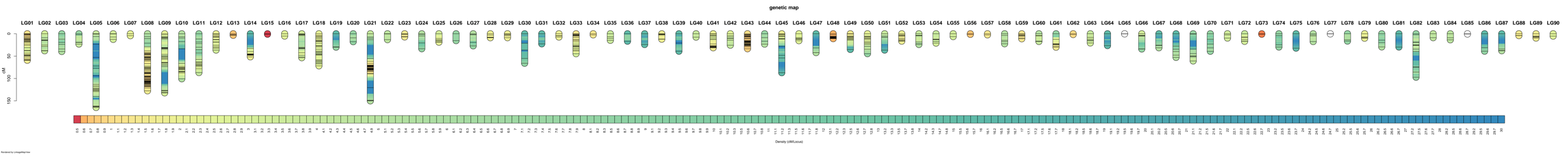

Supplement: Supplementary Figure 1 — Marker density of YT93-159 and ROC22 genetic linkage map. [file Data_Sheet_1.PDF]

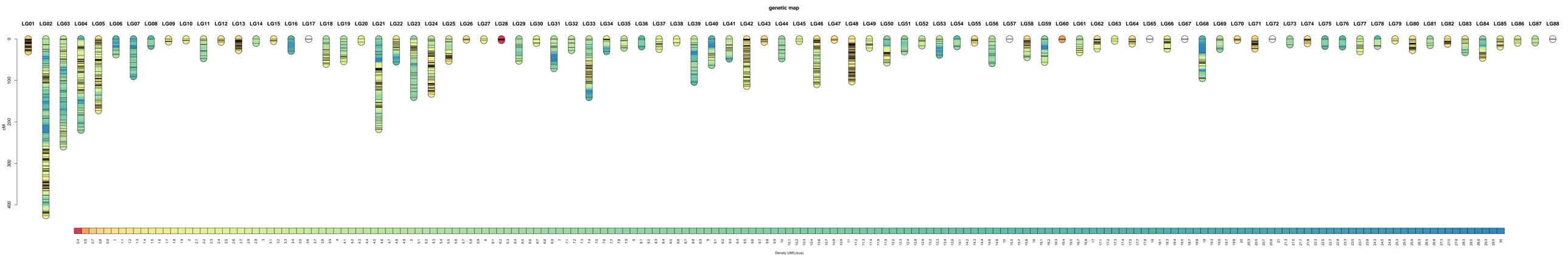

Supplement: Supplementary file 2 [file Data_Sheet_2.PDF]
